# Editorial: Decision-Making in Youth Sport

**DOI:** 10.3389/fpsyg.2021.725543

**Published:** 2021-07-29

**Authors:** Ana Filipa Silva, José Afonso, Hugo Sarmento, Sixto González-Víllora, Juan Carlos Pastor Vicedo, Israel Teoldo da Costa, Hermundur Sigmundsson, Luca Paolo Ardigò, Filipe Manuel Clemente

**Affiliations:** ^1^N2i, Polytechnic Institute of Maia, Maia, Portugal; ^2^The Research Centre in Sports Sciences, Health Sciences and Human Development (CIDESD), Vila Real, Portugal; ^3^Centre for Research, Education, Innovation and Intervention in Sport, Faculty of Sport of the University of Porto, Porto, Portugal; ^4^University of Coimbra, Research Unit for Sport and Physical Activity, Faculty of Sport Sciences and Physical Education, Coimbra, Portugal; ^5^Department of Physical Education, Arts Education, and Music, Faculty of Education of Cuenca, University of Castilla-La Mancha, Cuenca, Spain; ^6^Department of Physical Education, Arts and Music, Faculty of Education of Albacete, University of Castilla-La Mancha, Albacete, Spain; ^7^Physical Education Department, Centre of Research and Studies in Soccer (NUPEF), Universidade Federal de Viçosa, Viçosa, Brazil; ^8^Department of Psychology, Norwegian University of Science and Technology, Trondheim, Norway; ^9^Reykjavik University, Reykjavik, Iceland; ^10^Department of Neurosciences, Biomedicine and Movement Sciences, School of Exercise and Sport Science, University of Verona, Verona, Italy; ^11^Escola Superior Desporto e Lazer, Instituto Politécnico de Viana do Castelo, Rua Escola Industrial e Comercial de Nun'Álvares, Viana do Castelo, Portugal; ^12^Instituto de Telecomunicações, Delegação da Covilhã, Lisbon, Portugal

**Keywords:** decision making, youth, sports, exercise, young

Understanding decision-making in team sports motivated us to propose a Frontiers topic with eight published articles. These present different approaches to decision-making research in sports, partially confirming the hidden variation of experimental approaches related to this topic.

Decision-making is a complex process that plays a determinant role in sports performance. Knowledge related to the quality and effectiveness of this process is still being acquired, both in research and in practice. This may justify the fact that many studies are still focused on describing the decisions made, which more than bridge the gap for practice and with training interventions for developing children and young athletes. Possibly, further interventions should be made in the future.

Expecting to welcome different approaches to decision-making in youth sports, we aimed to improve the science and the practice in this specific field. Some of the included articles were descriptive, others proposed new approaches for training, and some synthesized the available evidence about training interventions.

## Descriptive Studies

One of the articles in this Frontiers topic introduced a new instrument for testing tactics (Machado and Costa). The *TacticUP* video test assesses the perceptual-cognitive and decision-making skills of soccer players. In their paper, Machado and Costa showed its validation process and reliability values, which ranged from 0.622 to 1.0. This test assesses offensive and defensive skills in situations near and distant from the ball based on the core tactical principles of soccer. The authors showed practical applications of TacticUP as an instrument that improves our understanding of players' skills as they develop measure the effectiveness of training programs, generate individualized player profiles, and can be used for talent development and selection processes.

In a different approach, a study analyzed the effect of coaches' paternalistic leadership on youth athletes' organizational citizenship behavior (Li et al.). The reports revealed that authoritative leadership did not correlate with organizational citizenship behavior, while benevolent and moral leadership did. Despite that, across different age groups, the same leadership style had different impacts and manifestations. The authors (Li et al.) emphasized that some coaches are only good at training youth athletes of a specific age and that coaches acting within the same age group may induce different behaviors and performance among the athletes.

## Perspectives

The review of Czyz explores the relationships between decision making and motor learning, especially regarding the management of variability practice, bridging different domains of research into a coherent whole. Initially approaching the theme from a cognitivist perspective, the author explores how practice conditions affect reaction time and decision-making, emphasizing open-loop controlled movements. The need to analyze complex environments under considerable time constraints invites the adoption of variable practice conditions (although there may be some space for applications of constant practice conditions). Within variable practices, the author discusses practice scheduling (i.e., blocked and random practice). Manipulating the degree of contextual interference becomes paramount in the context of making accurate decisions under effective reaction times. At this point, cognitivist approaches are linked to the constraints-led approach and the interactions between performer, environment, and task. Different models of information processing are presented for each type of practice (e.g., variable and constant). This review (Czyz) provides an interesting framework that will surely help coaches and teachers implement better-informed learning strategies.

Although the holistic development of athletes in the physical, cognitive, and psychological spheres is essential, tactics influence the other components of the game. With this idea in mind, the main objective of the article by Petiot et al. was to compare the application of these learning psychology frameworks to the acquisition of tactical intelligence. First, a long-term period is necessary to place players at the center of the process. Next, experience, knowledge of rules, specific skills, and tactical intelligence can be developed using repeated situations or in a wide range of contexts. The literature demonstrates that when game forms are adapted to the needs and levels of the players, they improve their sport competence. Indeed, tactical behaviors should be based on the evolution of the players. On the other hand, coaches should consider one important variable during the learning process that determines not only the tactical intelligence but also psychological outcomes per the complexity of the game. In doing so, the coach can modify certain parameters of the games, such as the number of players or the area of the game.

In summary, each variable (e.g., coach's learning style, specific game situations) determines players' perceptions of the game and how they decide to allocate resources to solve tactical and technical problems. Flexibility and adaptation to specific needs, previous experiences and the skill level of players are essential to promoting effective decision-making during gameplay.

## Interventions

In original work, Tanae et al. investigated the effect of interacting with virtual partners/opponents on motor plans since interpersonal interaction has a powerful influence on human perception, action, and cognition. The authors compared three types of interactions (competition, cooperation, and observation) and two types of virtual partners/opponents (those engaged in optimal motor planning and those engaged in risk-averse motor planning). The main results underline the importance of competition for modulating suboptimal decision-making and optimizing motor performance. This work highlights the importance of adjusting the levels of computer opponents to improve performance in e-sports athletes and its conclusions may potentially be translated into sports.

Three systematic reviews were published about training interventions' effects in decision-making and tactical behavior. In a systematic review with meta-analysis that analyzed the effects of small-sided games on technical and tactical skills of youth and young soccer players, it was possible to identify which technical skills benefited the most from small-sided games vs. analytical training approaches (Clemente, Ramirez-Campillo, Castillo et al.; Clemente, Ramirez-Campillo, Sarmento et al.).

It was also highlighted that evidence related to tactical behaviors is absent and must be the focus of future research (Clemente, Ramirez-Campillo, Sarmento et al.).

The systematic review with a meta-analysis conducted by Clemente, Ramirez-Campillo, Castillo et al. testing mental fatigue's effects on players' performance during small-sided games showed no meaningful impact of mental fatigue on the total distance covered or technical execution level of soccer players. Since the main outcomes did not consider different physical demands or technical/tactical behavior, the evidence should not be generalized.

The focus of Silva et al.'s systematic review with meta-analysis was to assess the effects of training programs on the decision-making of youth team sports players. All six studies showed a beneficial effect of decision-making interventions on tactical behavior, though no effect on technical execution was detected. The effectiveness of improving tactical behavior was independent of the number of sessions that players completed. These results should be carefully interpreted due to the heterogeneity of the articles' overall methodological quality.

In sum, the articles published in this special issue provide a diversified, heterogeneous approach demonstrative of the richness of studying decision-making in youth sport, while highlighting how much there is still to explore.

## Author Contributions

All authors listed have made a substantial, direct and intellectual contribution to the work, and approved it for publication.

## Conflict of Interest

The authors declare that the research was conducted in the absence of any commercial or financial relationships that could be construed as a potential conflict of interest.

## Publisher's Note

All claims expressed in this article are solely those of the authors and do not necessarily represent those of their affiliated organizations, or those of the publisher, the editors and the reviewers. Any product that may be evaluated in this article, or claim that may be made by its manufacturer, is not guaranteed or endorsed by the publisher.

